# Neuroprotective Effect of Simvastatin via Inducing the Autophagy on Spinal Cord Injury in the Rat Model

**DOI:** 10.1155/2015/260161

**Published:** 2015-10-11

**Authors:** Kai Gao, Guannan Wang, Yansong Wang, Donghe Han, Jing Bi, Yajiang Yuan, Tianchen Yao, Zhanghui Wan, Haihong Li, Xifan Mei

**Affiliations:** ^1^Department of Orthopedics, First Affiliated Hospital of Liaoning Medical University, Jinzhou 121000, China; ^2^College of Pharmacy, Liaoning Medical University, Jinzhou 121000, China; ^3^Department of Neurobiology and Key Laboratory of Neurodegenerative Diseases of Liaoning Province, Liaoning Medical University, Jinzhou 121000, China

## Abstract

Simvastatin, an inhibitor of 3-hydroxy-3-methylglutaryl-coenzyme A reductase, is invariably used to treat cardiovascular diseases. Simvastatin has been recently demonstrated to have a neuroprotective effect in nervous system diseases. The present study aimed to further verify the neuroprotection and molecular mechanism of simvastatin on rats after spinal cord injury (SCI). The expression of Beclin-1 and LC3-B was evidently enhanced at postoperation days 3 and 5, respectively. However, the reduction of the mTOR protein and ribosomal protein S6 kinase p70 subtype (p70S6K) phosphorylation level occurred at the same time after SCI. Simvastatin significantly increased the expression of brain-derived neurotrophic factor (BDNF) and glial cell line-derived neurotrophic factor (GDNF). Meanwhile, immunofluorescence results indicated that the expression of chondroitin sulfate proteoglycan (CSPG) and caspase-3 protein was obviously reduced by simvastatin. Furthermore, Nissl staining and Basso, Beattie, and Bresnahan (BBB) scores showed that the quantity and function of motor neurons were visibly preserved by simvastatin after SCI. The findings of this study showed that simvastatin induced autophagy by inhibiting the mTOR signaling pathway and contributed to neuroprotection after SCI.

## 1. Introduction

Spinal cord injury (SCI) is a medical problem worldwide [1]. Secondary injury significantly affects SCI and is induced by several factors, such as inflammation, oxidative stress, apoptosis, and autophagy [2, 3]. Autophagy is an important process to maintain cellular homeostasis and protects against a variety of diseases in the central nervous system [4, 5].

Simvastatin is widely used as the first-line therapeutic drug to reduce cholesterol and prevent coronary heart diseases and atherosclerosis. In recent years, an increasing number of studies have investigated the pleiotropic effects of statins, such as anti-inflammation, immunoregulation, and the induction in autophagy [6, 7]. Stüve et al. [8, 9] demonstrated that simvastatin has an effective therapeutic effect on multiple sclerosis, Alzheimer's disease, ischemic stroke, and rheumatoid arthritis. However, clinical observation showed that these effects cannot be attributed to the beneficial aspects of cholesterol-lowering activity [10].

Recent studies have shown that autophagy is activated by simvastatin through inhibiting the mTOR signaling pathway, which alleviates myocardial cell damage, promotes regeneration of myocardial cells, and prevents the occurrence of atherosclerosis [11]. Simultaneously, some reports also indicated that simvastatin promotes functional recovery after SCI by lowering the activity of astrocytes and the expression of inflammatory factors [12–14]. However, the molecular mechanism of the neuroprotection after SCI has not been elucidated.

In the present study, the effect of simvastatin on autophagy after SCI is initially proposed. We attempted to provide evidence that further validates the neuroprotection of simvastatin and investigate the molecular mechanism of the neuroprotection after SCI.

## 2. Materials and Methods

### 2.1. Animal Care and Groups

Eighty-one adult male Sprague-Dawley rats (240–260 g) were purchased from the Laboratory Animal Center of the Liaoning Medical University. The rats were raised in the SPF laboratory animal center at constant environment of 23 ± 0.5°C, with an alternating 12 h light-dark cycle. All experimental procedures were carried out in accordance with the Guidelines for the Care and Use of Laboratory Animals published by the US National Institutes of Health. All efforts were made to minimize the number of animals used and their suffering.

The rats were randomly divided into three groups: (1) simvastatin, (2) vehicle, and (3) sham. Simvastatin (10 mg/kg) and vehicle were administered via intraperitoneal injection at 1 h after SCI and then once daily for 2 days [6, 12].

### 2.2. SCI

The rats were operated on as previously described [15]. In brief, the rats were anesthetized with intraperitoneal injection of chloral hydrate (0.33 mL/kg) and the spinal cord was aseptically exposed after laminectomy at T9-10. Subsequently, a 2 mm diameter impounder (weight: 10 g) was dropped from a height of 25 mm and struck at the surface of T9-10 spinal cord, which led to spinal cord congestion. The sham-operated animals underwent laminectomy alone. After SCI, manual bladder expression was performed thrice daily until the bladder function of the rats was reestablished.

### 2.3. Simvastatin Treatment

Simvastatin (Catalog number 567020; Calbiochem, Germany) was dissolved in 100% ethanol and activated by 0.1 N NaOH after heating at 50°C for 2 h. pH was then adjusted to 7.2, and stock solution was maintained at 4°C prior to use [16, 17].

### 2.4. Locomotion Recovery Assessment

The Basso, Beattie, and Bresnahan (BBB) open-field locomotor rating scale was employed to evaluate the recovery condition of motor function after SCI [18]. In brief, three independent examiners were blinded to assess the BBB scores before operation and at 1, 3, 7, 14, 21, 28, and 35 d after SCI. The BBB scores ranged from 0 to 21 points. The minimum points (0) indicated complete paralysis, and the maximum points implied normal function. The average scores were calculated according to the progress in locomotion recovery after SCI.

### 2.5. Western Blot Analysis

At 3, 5, and 7 d after SCI, the rats were anesthetized, and the damaged spinal cord (2 mm cephalad and caudally around the injury epicenter) was removed. The tissues were dissolved in RIPA lysis buffer, and the final protein concentration (2 *μ*g/*μ*L) was quantified using the BCA kit. Protein samples (40 *μ*g) were then emitted into different lanes. The proteins were separated by SDS-PAGE and transferred into PVDF membranes. Subsequently, the membranes were incubated at 4°C overnight with primary antibodies including anti-mTOR antibody (1 : 1000; Novus Biologicals, USA), anti-phospho-p70SK antibody (1 : 500; Abcam, Cambridge, UK), anti-LC3 antibody (1 : 1000; Novus Biologicals, USA), anti-Beclin-1 antibody (1 : 1000; Abcam), anti-brain-derived neurotrophic factor (BDNF) antibody (1 : 1000; Novus Biologicals, USA), antiglial cell line-derived neurotrophic factor (GDNF) antibody (1 : 1000; Novus Biologicals, USA), and anti-*β*-actin antibody (1 : 1000; Abcam, Cambridge, UK). On the second day, the membranes were incubated at room temperature for 2 h with secondary antibodies (1 : 2000; Abcam, Cambridge, UK). The membranes were developed using ChemiDoc-It TS2 Imager (UVP, LLC, Upland, CA, USA), and relative optical density was performed using ImageJ2x software (National Institutes of Health, Bethesda, MD, USA).

### 2.6. Immunofluorescence Analysis

The rats were anesthetized and perfused with 0.9% saline and then with 4% paraformaldehyde at 1 week after SCI. The T8–T12 segments of the spinal cord were excised from the rats and steeped into 4% paraformaldehyde. The 5 *μ*m crosswise sections (3 mm rostral to the epicenter) were cut using a cryostat microtome. The sections were dried at room temperature and were placed into 0.01 M citric acid (pH 6.0) for antigen retrieval. Next, the sections were blocked with blocking buffer (5% normal goat serum and 0.1% Triton X-100 in PBS) at 4°C for 1 h and incubated overnight with primary antibodies including anti-CSPG antibody (1 : 400; Sigma-Aldrich) and anti-caspase-3 antibody (1 : 100; Novus Biologicals, USA). The following day, the sections were incubated with FITC goat anti-rabbit/mouse IgG, and the nucleus was redyed with DAPI solution. All slides were observed under a fluorescence microscope (Leica, Heidelberger, Germany) after being mounted with Permount TM mounting medium (Sigma-Aldrich, St. Louis, MO, USA). The optical density of fluorescence was analyzed using ImageJ2x software.

### 2.7. Nissl Staining

The rats were perfused with 0.9% saline and 4% paraformaldehyde at 7 d after SCI. The 20 *μ*m crosswise sections (3 mm rostral to the epicenter) were cut using a cryostat microtome. The sections were dried and then soaked directly into the mixed liquor (1 : 1 alcohol/chloroform) overnight. The sections were successively rehydrated with 100% alcohol, 95% alcohol, and distilled water. Subsequently, the sections were stained in 0.1% Cresyl violet (Sigma-Aldrich) solution. The sections were then differentiated in 95% ethyl alcohol, dehydrated in 100% alcohol, and rinsed in xylene. Finally, the sections were mounted and observed under a light microscope. The average quantity of neurons was calculated by randomly selecting five Nissl-stained sections at the same site from each rat.

### 2.8. Statistical Analysis

All data were expressed as mean ± SD and analyzed using the Graph Prism Program, Version 5.0 (GraphPad Software, Inc., La Jolla, USA). Unpaired Student's *t*-test was used for the comparison among groups. The discrepancy of the multiple groups was tested using one-way ANOVA, and the BBB scores were analyzed with the Mann-Whitney *U* test. *p* values less than 0.05 were considered statistically significant, and the discrepancy was statistically significant if *p* < 0.01.

## 3. Results

### 3.1. Simvastatin Improves Motor Functional Recovery after SCI

**Figure 1:**
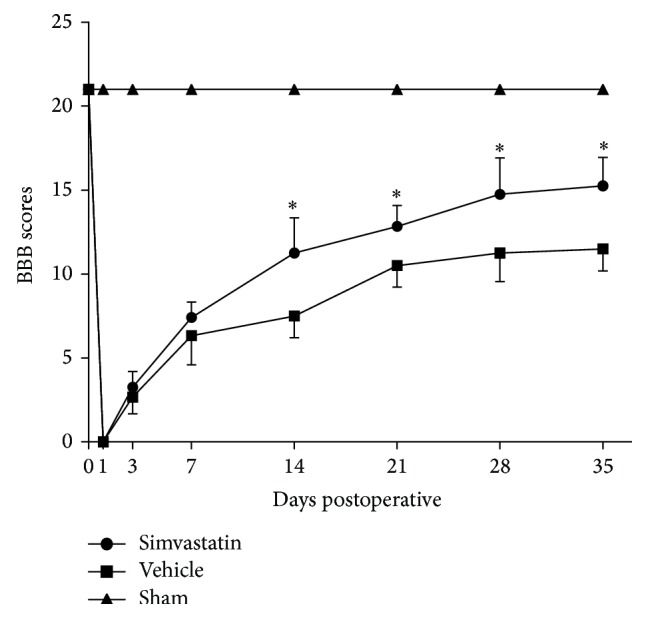
The BBB scores was evaluated at 0, 1, 3, 7, 14, 21, 28, and 35 d severally for observing the recovery situation of motor functional after SCI. Data are means ± SD of 6 rats for each group. (_ _^*∗*^ *p* < 0.05 compared with vehicle group).

We assessed the locomotor performance of rats by using the BBB locomotor rating scale at 0, 1, 3, 7, 14, 21, 28, and 35 d. The curve graph shows the tendency to change based on the scores (Figure 1). We found that the locomotor function was drastically reduced in the first day after SCI and gradually recovered with time, whereas the resumptive levels were distinct between the simvastatin and vehicle groups. At 3 and 7 d, the recovery of motor function in the simvastatin group was similar to the vehicle group (*p* > 0.05). Nevertheless, at 14 d after SCI, the simvastatin-treated rats (BBB scores: 11.3 ± 2.1) showed a more significant recovery than the vehicle group (BBB scores: 7.5 ± 1.3; *p* < 0.05). Similarly, the significant tendency also increased at 21, 28, and 35 d (BBB: 21 d after operation: simvastatin group (12.8 ± 1.3) compared with the vehicle group (10.5 ± 1.3); 28 d after operation: simvastatin group (14.8 ± 2.2) compared with the vehicle group (11.3 ± 1.7); and 35 d after operation: simvastatin group (15.3 ± 1.7) compared with the vehicle group (11.5 ± 1.3); *p* < 0.05). The trend revealed that the motor function of the rats is increasingly restored, and recovery was faster after 14 d with simvastatin treatment.

### 3.2. Simvastatin Induces Autophagy by Inhibiting the mTOR Signaling Pathway after SCI

**Figure 2:**
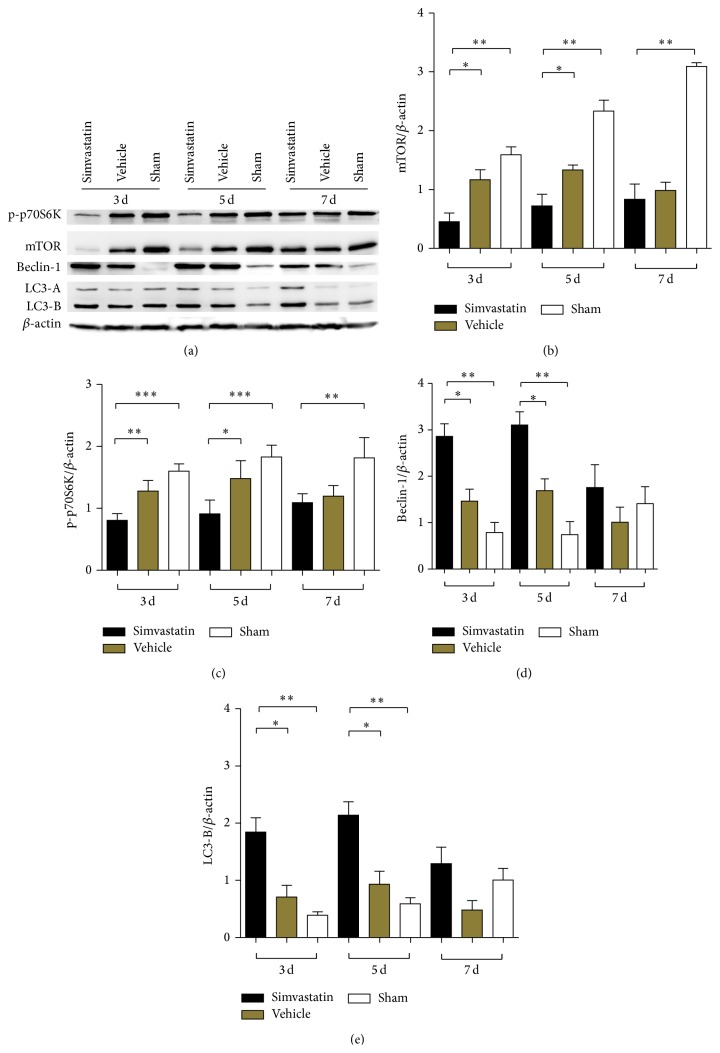
The western blot was utilized to detect the expression of mTOR, p-p70S6K, Beclin-1, and LC3-B at 3, 5, and 7 d after SCI (a). (b), (c), (d), and (e), respectively, showed the average relative gray of mTOR, p-p70S6K, Beclin-1, and LC3-B compared to the *β*-actin protein. Data are expressed as the mean ± SD (*n* = 4/group, _ _^*∗*^ *p* < 0.05, _ _^*∗∗*^ *p* < 0.01, _ _^*∗∗∗*^ *p* < 0.001).

Western blot detected the expression of Beclin-1, LC3, mTOR, and p-p70S6K proteins for observing the alteration of autophagy in the three groups (Figure 2(a)). Upon formation of the autophagy membrane, conversion from the nonlipidated form (LC3-A) to the lipidation form (LC3-B) is considered to be an important symbol of autophagic activation. LC3-B is the form incorporated in autophagosomes and thus often used as a marker for autophagosomes [19–21]. At the same time, Beclin-1 protein is commonly used for autophagy detection, which is a unique autophagy-related protein [22]. The expression levels of Beclin-1 and LC3-B proteins significantly increased in the presence of simvastatin compared with the absence of simvastatin at 3 and 5 d (Figures 2(d) and 2(e)). However, the results were not statistically different at 7 d after SCI (Figures 2(d) and 2(e), *p* > 0.05 compared with the vehicle). In addition, to examine whether simvastatin suppressed the mTOR signal pathway, western bolt showed that the expressions of mTOR protein and p70S6K phosphorylation level were distinctly lower in the simvastatin-treated group in contrast to the vehicle and sham groups at 3 and 5 d (Figures 2(b) and 2(c)). Similarly, the statistical difference was not evident at postoperation day 7 (Figures 2(b) and 2(c), *p* > 0.05 compared with the vehicle group).

### 3.3. Expression of BDNF and GDNF Is Upregulated by Simvastatin after SCI

**Figure 3:**
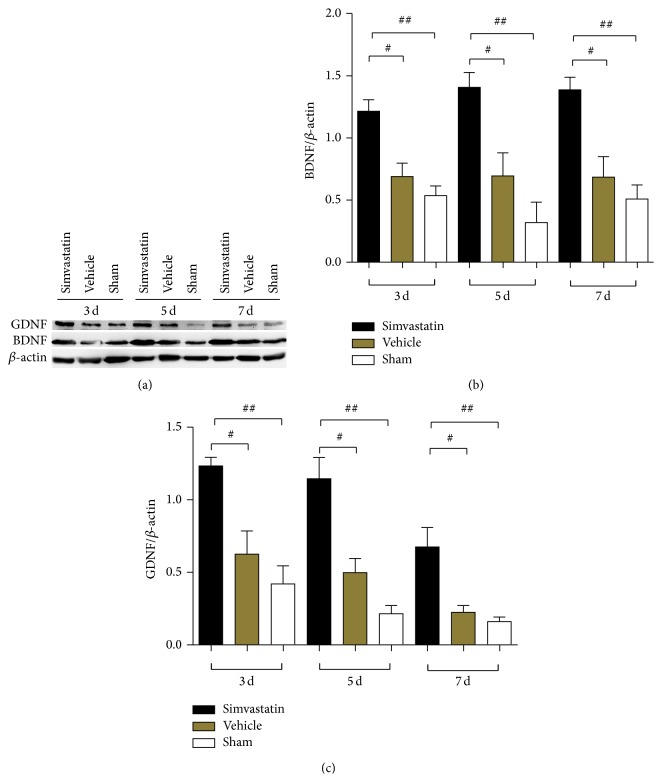
The effect of simvastatin on the BDNF and GDNF expression at 3, 5, and 7 d after SCI (a). The expression levels of BDNF and GDNF were markedly improved at 3, 5, and 7 d, respectively, in the presence of simvastatin compared with the absence of simvastatin (b) and (c). All experiments were repeated 4 times and ^#^ *p* < 0.05; ^##^ *p* < 0.01.

The spinal cord tissues were obtained from the rats at 3, 5, and 7 d after SCI for the western blot to detect the expression of BDNF and GDNF (Figure 3(a)). The consequences revealed that simvastatin treatment significantly improved the expression levels of BDNF and GDNF after injury. By contrast, the two neurotrophic factors were weak in the vehicle and sham groups at the same time (Figures 3(b) and 3(c): *p* < 0.05 compared with the vehicle group; *p* < 0.01 compared with the sham group).

### 3.4. Simvastatin Decreases the Expression of CSPG Protein

**Figure 4:**
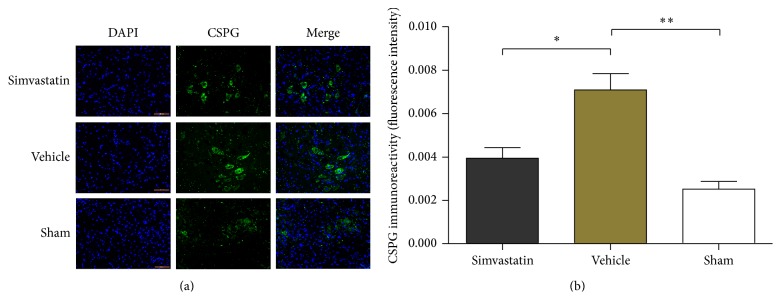
The immunoreactivity of CSPG by the immunofluorescence analysis in the three groups (a); the expression of CSPG protein in the presence of simvastatin compared to the vehicle treatment (b). Data are means ± SD (*n* = 4/group, _ _^*∗*^ *p* < 0.05, _ _^*∗∗*^ *p* < 0.01).

Immunofluorescence assay was performed to observe the CSPG expression level at 1 week after SCI (Figure 4(a)).  Figure 4(b) shows that compared with the vehicle group, the relative staining intensity of CSPG was dramatically reduced by simvastatin after SCI.

### 3.5. Apoptosis Is Inhibited by Simvastatin after SCI

**Figure 5:**
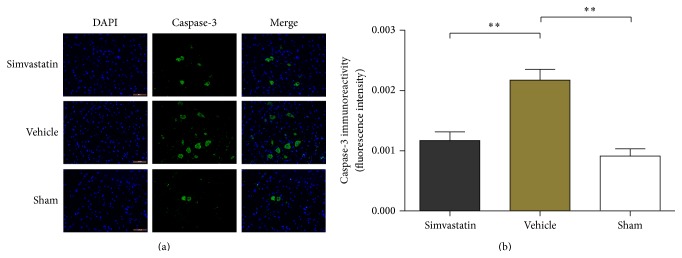
The condition of apoptosis was reflected by the expression level of caspase-3 in the three groups (a); the expression of caspase-3 in the rats with the different treatment after SCI (b). Data are means ± SD (*n* = 4/group; scale bar: 100 *μ*m, _ _^*∗∗*^ *p* < 0.01).

Immunofluorescence analysis was used to detect the staining intensity of caspase-3 at 7 d after SCI (Figure 5(a)). In comparison to the sham group, the expression level of caspase-3 was clearly upregulated after SCI. Nevertheless, simvastatin treatment evidently decreased the expression level of caspase-3 after SCI (Figure 5(b)), indicating that simvastatin treatment obviously inhibited the apoptosis after SCI.

### 3.6. Simvastatin Reduces the Loss of Nissl Bodies in Rats after SCI

**Figure 6:**
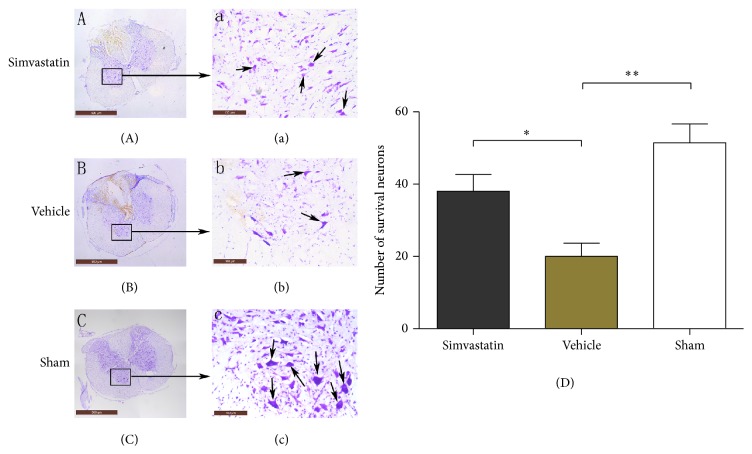
Survival of neurons in the spinal cord at postoperative week 1 was shown by Nissl staining (A–D). (Scale bar: (A), (B), and (C), 500 *μ*m; (a), (b), and (c), 100 *μ*m; *n* = 5/group, _ _^*∗*^ *p* < 0.05, _ _^*∗*^ *p* < 0.01).

Nissl staining showed that the quantities of Nissl bodies in the rat spinal cord were evidently reduced in the vehicle group after SCI compared with the sham group. By contrast, compared with the saline group, the injury was distinctly improved and the damaged tissue was also clearly reduced by simvastatin (Figure 6(A), (a); (B), (b); and (C), (c)). Meanwhile, the quantities of motor neurons in the anterior horns were significantly increased by simvastatin compared with the vehicle group (Figure 6(D), *p* < 0.05).

## 4. Discussion

Statins are first-line therapeutic drugs that reduce cholesterol and prevent coronary heart disease and atherosclerosis. Recently, the multiple effects of simvastatin, including anti-inflammation and immunoregulation and, especially, the positive effects on the central nervous system, have attracted more researchers [12, 23]. Favorable effects of simvastatin on the nervous system diseases are accepted. However, according to some scholars, simvastatin failed to improve the functional recovery after SCI [24, 25]. In the present study, the optimum dose of simvastatin (10 mg/kg) was selected based on previous reports [11, 12, 16]. In addition, the molecular mechanism of simvastatin regulating autophagy after SCI was initially observed in this study. The study may further sustain the views that simvastatin has an underlying neuroprotective effect and may promote the progress of clinical research of simvastatin for treating SCI.

Over the past few years, autophagy has played an important role in the process of motor functional recovery after SCI [26, 27]. Autophagy has a significant effect in many neurodegenerative diseases as well as in traumatic and ischemic brain injuries [28]. Meanwhile, the mTOR signaling pathway plays a significant role in activating autophagy. Other reports also demonstrated that the mTOR signaling pathway has a significant function in neurodegenerative diseases, such as Alzheimer's disease, Parkinson's disease, Huntington's disease, and SCI [29, 30].

Previous studies considered that autophagy is strengthened via inhibiting the mTOR signaling pathway in coronary arterial myocytes and colchicine-induced muscle toxicity with simvastatin treatment [6, 11, 31]. However, no relevant reports exist about the effect of simvastatin on autophagy after SCI. In the present study, western blot results exhibited that the expression levels of Beclin-1 and LC3-B proteins were significantly increased at 3 and 5 d by simvastatin after SCI. By contrast, the p70S6K phosphorylation level (the downstream effector of mTOR signaling) [29, 32] and the mTOR protein level were significantly inhibited by simvastatin. Results revealed that autophagy was significantly activated by inhibiting the mTOR signaling pathway after SCI with simvastatin treatment, consistent with the effect of simvastatin on autophagy in other diseases and reports [11, 33, 34]. Interestingly, our results also demonstrated that the alteration of Beclin-1, LC3-B, mTOR, and p-p70S6K expression level was not statistically significant at 7 d in the simvastatin-treated group compared with the vehicle-treated group after SCI. Therefore, a novel hypothesis was proposed that autophagy is likely to be evidently influenced by simvastatin in the acute phase of SCI. Thus, the role of simvastatin in autophagy in the different phases of SCI needs further study.

The inhibition of axon regeneration generates unfavorable recovery environment for neurological function and restricts the functional rehabilitation after injury in the central nervous system. Some researchers reported that CSPG occupies a consequential restrictive effect on axonal growth and regeneration [14, 35]. In addition, numerous studies also show that the neurotrophic factors BDNF and GDNF play important roles in neuronal survival process, improving the remyelination of injured axons and neuronal regeneration after SCI [36–38]. In the current study, the influence of simvastatin on the expression of CSPG, BDNF, and GDNF was assessed using immunofluorescence and western blot analysis. The results demonstrated that simvastatin treatment had the potential for neurological functional recovery after SCI.

Increasing evidence has shown that apoptosis plays a critical role in the diseases of nervous system, such as Alzheimer's disease and SCI [39]. The presence of apoptosis imposes restrictions on the neural functional recovery. The low expression of caspase-3 protein, a critical regulator of apoptosis, indicates that the activity of apoptosis is inhibited, which plays a neuroprotective role in the central nervous system [40]. In present study, the immunofluorescence results revealed that simvastatin apparently downregulated the expression of caspase-3. This downregulation of expression implies that apoptosis was inhibited and neurological function may be ameliorated by simvastatin.

To further confirm our hypothesis that simvastatin could improve neurological function, we used Nissl staining and BBB scores to observe the functional rehabilitation of motor neurons in different groups. The vehicle group showed that the quantity and function of motor neurons were visibly improved by simvastatin. The study results validated our hypothesis that simvastatin exhibits neuroprotection on SCI. Significantly, in our current study, the neuroprotective effect of simvastatin has been further verified on SCI, which is consistent with other reports [12, 13, 16]. Furthermore, a novel molecular mechanism of neuroprotection of simvastatin may have also been discovered after SCI and the significant results may provide a potential of clinical application of simvastatin for treating SCI.

## 5. Conclusions

Simvastatin induced autophagy by inhibiting the mTOR signaling pathway and had the potential for neuroprotection after SCI. However, further verification on the comprehensive effects and molecule mechanism of simvastatin on animals and various nerve cells after injury is necessary.
